# Proton Binding Characteristics of Dissolved Organic Matter Extracted from the North Atlantic

**DOI:** 10.1021/acs.est.3c01810

**Published:** 2023-12-05

**Authors:** Pablo Lodeiro, Carlos Rey-Castro, Calin David, Matthew P. Humphreys, Martha Gledhill

**Affiliations:** † Department of Chemistry, Physics, Environmental and Soil Sciences, University of Lleida − AGROTECNIO-CERCA Center, Rovira Roure 191, 25198 Lleida, Spain; ‡ Department of Ocean Systems (OCS), NIOZ Royal Netherlands Institute for Sea Research, P.O. Box 59, 1790 AB Den Burg (Texel), The Netherlands; § GEOMAR Helmholtz Centre for Ocean Research Kiel, Wischhofstraße 1-3, 24148 Kiel, Germany

**Keywords:** DOM, nonideal competitive adsorption (NICA), Donnan, proton binding, acid−base, log *K̅*_H_, solid−phase extraction, seawater; heterogeneity

## Abstract

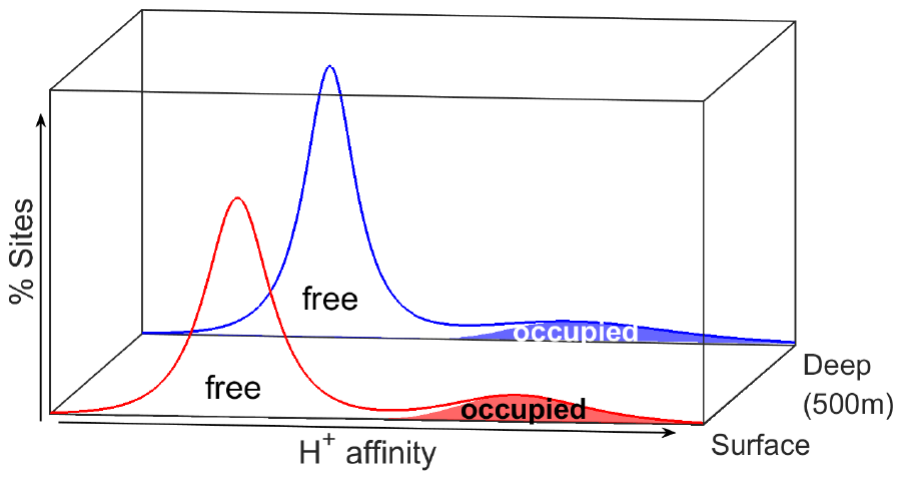

Marine dissolved organic matter (DOM) presents key thermodynamic properties that are not yet fully constrained. Here, we report the distribution of binding sites occupied by protons (i.e., proton affinity spectra) and parametrize the median intrinsic proton binding affinities (log *K̅*_H_) and heterogeneities (*m*), for DOM samples extracted from the North Atlantic. We estimate that 11.4 ± 0.6% of C atoms in the extracted marine DOM have a functional group with a binding site for ionic species. The log *K̅*_H_ of the most acidic groups was larger (4.01–4.02 ± 0.02) than that observed in DOM from coastal waters (3.82 ± 0.02), while the chemical binding heterogeneity parameter increased with depth to values (*m*_1_= 0.666 ± 0.009) ca. 10% higher than those observed in surface open ocean or coastal samples. On the contrary, the log *K̅*_H_ for the less acidic groups shows a difference between the surface (10.01 ± 0.08) and deep (9.22 ± 0.35) samples. The latter chemical groups were more heterogeneous for marine than for terrestrial DOM, and *m*_2_ decreased with depth to values of 0.28 ± 0.03. Binding heterogeneity reflects aromatic carbon compounds’ persistence and accumulation in diverse, low-abundance chemical forms, while easily degradable low-affinity groups accumulate more uniformly in the deep ocean.

## Abstract 2

The thermodynamic binding data presented here for marine DOM are required for a robust description of the current marine carbon cycle and predictions under ongoing climate change.

## 1 Introduction

Dissolved organic matter (DOM) comprises the largest pool of organic carbon in seawater (ca. 660 Pg of C) in which it plays important roles.^1,2^ The bioavailability and toxicity of trace metals in the ocean depend on their chemical speciation, which is the result of environmental conditions and interactions between trace elements and organic matter.^3^ The competition effects between chemical species for binding to marine DOM and the overall binding capacity are also related to the DOM intrinsic acid–base/proton binding properties,^4,5^ which define organic alkalinity.^6^ Understanding the interdependencies between the intrinsic binding properties of marine DOM and the biogeochemical cycling of trace metals, along with the contribution of organic matter to total alkalinity by interacting with protons, is therefore of high relevance to the contemporary ocean and future climate change scenarios.

The composition of marine DOM can be expected to influence the magnitude and distribution of the intrinsic (i.e., chemical) ion binding affinities. The molecular composition of marine DOM suggests lower diversity and less aromaticity than terrestrial organic matter.^7^ Nevertheless, the molecular complexity of marine DOM^8^ means that marine DOM will not behave as a well-characterized simple acid molecule (e.g., acetic acid) with discrete values for the proton binding affinities, as most often described by complexometric titrations in seawater.^9^ Rather, ion binding affinities occur over a continuum of values (i.e., affinity spectra) and fall into groups comprised of different organic acids with a variable range of binding affinities, as is typically observed for terrestrial organic matter.^10^ This variable interval of affinity values is related to the chemical binding heterogeneity, which is a consequence of the compositional heterogeneity that describes DOM at the molecular level.^11^ Indeed, a potential correlation between ion binding affinity and molecular composition of DOM is expected, although this topic is beyond the scope of this manuscript.

The chemical binding heterogeneity of open ocean marine DOM reflects its complex nature but is a key parameter not yet constrained. This gap in our current knowledge about the proton binding behavior of marine DOM is relevant to the often observed inconsistencies between measured and calculated parameters of the carbonate system in the ocean (e.g., pH or total alkalinity), which are partially due to the lack of a complete thermodynamic description of the acid–base chemistry of seawater.^12,13^

The binding of trace metals and protons to marine organic ligands is often described using simplified conditional stability constants.^14,9^ However, for a better understanding of trace element biogeochemistry, it is essential to untangle the main drivers that influence chemical speciation (e.g., pH, ionic strength and temperature) and determine a complete set of intrinsic binding parameters for marine DOM, which are thermodynamically consistent and independent from the specific conditions of the seawater sample.^15^

Here we present experimental results for open ocean environments, based on the solid-phase extractable fraction of DOM (ca. 38% DOC recovery yield), obtained from proton binding titrations of samples from surface (ca. 2 m depth) and deep (500 m depth) waters of the North Atlantic, for the first time. We determine the intrinsic binding properties of the main chemical groups involved in the binding of cations to oceanic DOM following well-established procedures. We used a combination of the nonideal competitive adsorption (NICA) isotherm, which describes the chemical binding to heterogeneous ligands, and the Donnan electrostatic model for the description of polyelectrolytic effects, i.e., the nonspecific binding. Finally, we critically compare the calculated intrinsic proton binding parameters of North Atlantic DOM with values from the semienclosed Baltic Sea^5^ and from generic freshwater organic matter.^16^

## 2 Materials and Methods

### 2.1 Seawater Collection

Samples were collected in the North Atlantic during the *Meteor* cruise GApr11 (M147 Amazon–GEOTRACES). A water sample (total 470 L) was collected from between 16°25.977′ – 10°07.155′ N and 28°47.427′ – 36°04.983′ W (see Figure S1) on the 24th of April 2018 while the ship was underway via a Teflon bellows pump (Almatec A15) and acid-washed tubing suspended at ca. 2 m depth and ca. 4 m distance from the ship using a towed fish. Water was filtered through 0.8/0.2 μm cartridge filters (AcroPak1000), pumped into water sampling bottles (24 × 12 L, CFree, Ocean Test Equipment) placed inside a trace metal–clean laboratory container, and then acidified with HCl (Romil UHP grade) to a final pH of 2 prior to DOM preconcentration. Deep water samples were collected at ca. 500 m depth from two locations: 09°29.91′ N, 036°47.524′ W and 04°09.524′ N, 042°54.72′ W (see Figure S1), on the 25th and 27th of April 2018, respectively. A total of 460 L was collected from the water sampling bottles deployed on an epoxy-coated aluminum rosette equipped with a Seabird SBE 911 plus CTD. The collected deep seawater was acidified prior to DOM preconcentration as done for the surface water.

### 2.2 Seawater Analysis

Sampling and methods of analysis for macronutrients (phosphate, silicic acid, nitrate, and nitrite), dissolved organic carbon (DOC), total dissolved nitrogen (TDN), pH, temperature, and salinity at sampling points are described in detail elsewhere.^17^

### 2.3 Dissolved Organic Matter Preconcentration and Extraction

Dissolved organic matter was preconcentrated from the collected seawater using solid–phase extraction cartridges, Mega Bond–Elut Priority PolLutant (PPL) 5 g, 60 mL from Agilent. The PPL cartridges had previously been soaked in 50 mL of methanol (Fisher Scientific LC–MS grade) for 12 h and then washed by passing 15 mL of HCl (0.1% v/v) through each cartridge before use. The seawater was passed through the PPL cartridges with a slight overpressure provided by filtered nitrogen gas (99.999%, AlphaGaz). One cartridge was used for every 20 (surface) or 29 (depth) L of seawater. Maximum cartridge loading was ca. 1.5 mg of C per gram of PPL. Afterward, the PPL cartridges were stored frozen (−20 °C). For the DOM extraction, the cartridges were defrosted at room temperature, then washed with 15 mL of ultrapure water, and soaked with (2×) 10 mL of acetonitrile for 10 min to elute the DOM. The acetonitrile–DOM solution (20 mL from each cartridge) was collected in a Teflon pot and dried under a stream of ultrapure N_2_ gas. The extraction efficiencies were determined as the ratios between the DOC content of the DOM extracts and the DOC content in the original seawater samples.

### 2.4 Dissolved Organic Matter Stock Solutions

The solid DOM extracts were dissolved in NaOH (∼0.02 M, extra pure, 98%, Acros Organics) to final solid-phase extracted dissolved organic matter (SPE-DOM) concentrations of 5.15 and 6.79 g·L^–1^ for surface and deep samples, respectively, and preserved in the dark at 4 °C. These SPE–DOM stock solutions were used, usually within a week of preparation, to obtain the samples for titration, as described below.

### 2.5 Potentiometric Titrations

A detailed description of the experimental setup and conditions was provided by Lodeiro et al.^4^ Here, DOM concentrations in the titration vessels were 1.03 g of DOM·L^–1^ (366 mg C·L^–1^) and 1.18 g of DOM·L^–1^ (345 mg C·L^–1^) for surface and deep samples, respectively. The relatively high concentration of DOM used in the titration experiments is motivated by the low percentage of C (29–35%) and low number of titratable groups per C atom (0.112–0.117 mol·molC^–1^, see Table S3) in our extracted DOM. The ionic strength (I) of the titrated solutions was fixed to values of 0.007, 0.1, 0.7, and 1 M using NaCl (puriss. p.a., ≥ 99.5%, Merck) as an inert background electrolyte. We prepared calibration solutions with the same ionic strengths as the samples and defined the pH and log *K̅*_H_ on the free proton concentration scale. The conversion to e.g., pH(NBS) can easily be carried out using a suitable ion activity coefficient for H^+^ in seawater.^18^ A typical SPE–DOM titration experiment took about 6–8 h, including an initial solution equilibration step under N_2_ bubbling of ca. 1 h. These long equilibration times were the result of a strict stabilization criterium of the mV readings of the glass electrode (<0.05 mV/min), the high concentration of the DOM in the titration vessel, and the experimental limitations of the glass electrode at pH values above 8.5–9.5.

### 2.6 The NICA–Donnan Model

The proton titration data was described by the bimodal NICA isotherm and the Donnan electrostatic model.^19−22^ In the absence of metal cations able to compete with protons for the specific binding to the functional groups of DOM (monocomponent system), the bimodal NICA isotherm is formally identical to the weighted sum of two Langmuir–Freundlich isotherms^10^
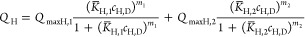
1where *Q*_H_ stands for the amount of bound protons per mol of DOC (mmol·mol C^–1^), *Q*_maxH,*j*_ is the total amount of titratable proton binding sites within each distribution, *c*_H,D_ is the proton concentration in the Donnan phase, *K̅*_H,*j*_ is the median value of the *j*^th^ intrinsic proton binding affinity distribution, and *m*_*j*_ (0 < *m*_*j*_ ≤ 1) is related to the width of the affinity distribution function (a measure of the chemical binding heterogeneity). The limiting value of *m*_*j*_ = 1 corresponds to a perfectly homogeneous set of sites. The subindexes for *j* = 1 or 2 represent the two main groups in the affinity distribution of binding sites, hereafter called DOM_1_ and DOM_2_. The group with the lowest affinity for protons (DOM_1_) comprises the most acidic sites, usually associated with carboxylic-like functional groups, whereas the high affinity group DOM_2_ includes the less acidic sites often associated with phenolic-like chemical groups.

The Donnan model was used to describe the electrostatic contribution to the effective ion binding by DOM. The Donnan model considers that DOM is an electroneutral, permeable gel phase with a homogeneous distribution of fixed charges that originate from dissociation of the binding groups.^20,23^ We followed the “master curve approach” and used the gel phase (Donnan) volume (*V*_D_) as a variable to fit the obtained charge curves. We used an expression for *V*_D_ consistent with the nonlinear Poissson-Boltzman (NLPB) equation (PB-*V*_D_), which depends on both the ionic strength and macromolecular charge, for the description of the electrostatic binding to marine DOM.^24^ In addition, we also provide binding parameters using a standard *V*_D_ equation, an empirical expression where *V*_D_ depends only on ionic strength^23^ for comparison. Although more refined electrostatic models are available in the recent literature,^25^ they rely on detailed molecular information (such as molecular weights and particle radius) that is not currently available, so this topic will be worthy of future research. A detailed description of the Donnan approach models with the PB-*V*_D_ and standard expression for *V*_D_ used in this work can be found in Lodeiro et al.^4^

The strategy followed to derive the NICA-Donnan model parameters can be found in the Supporting Information. Briefly, the experimental titration data, *E* (mV) vs *V*_NaOH_ (mL), were converted to apparent charge curves, (*q*_DOM_ = *Q*_maxH,tot_ – *Q*_H_) vs pH, using mass and charge balance relationships and the calibration of the electrode in the free proton concentration scale at each ionic strength. The fitting of the NICA-Donnan model to these (pH, *Q*_maxH,tot_ – *Q*_H_) curves was carried out by iteratively solving the set of equations of the NICA expression for proton binding and the electrostatic model, as detailed in the Supporting Information of Lodeiro et al.^4^ Finally, the optimized model parameters were obtained by minimizing the sum of squared residuals between the experimental and theoretical charges.

## 3 Results and Discussion

### 3.1 Ancillary Parameters

The values observed for salinity, DOC, pH, oxygen, and nutrient concentrations in the surface and deep North Atlantic samples were in line with profiles previously reported for these locations^26^ (Tables S1 and S2). We extracted 0.515 g (surface) and 0.340 g (depth) of DOM after preconcentrating 460–470 L of seawater, with an equivalent DOC recovery of 38.4 ± 2.0% for surface and 37.9 ± 4.2% for deep samples. These recovery values are lower than those usually obtained in fresh and brackish waters,^27^ and some North Atlantic deep samples,^7,26^ but in agreement with other previous yields of DOM from the open ocean and Mediterranean Sea^29−32^ using PPL cartridges. Note that the extraction procedure may introduce a bias in the DOM composition,^33^ which could affect the number and type of chemical compounds analyzed in our titration experiments.

The organic C/N molar ratios for surface and deep North Atlantic seawater samples were 13.2 ± 3.7 and 17.7 ± 2.9, respectively. These values, and the observed increase of the C/N molar ratio with depth, are in agreement with open ocean ratios previously reported and indicate preferential remineralization of N from DOM as it is transported into deeper waters.^34,35^ The extracted DOM samples showed C/N molar ratios higher than those of the bulk seawater. The isolated surface DOM solution had a C_SPE_/N_SPE_ molar ratio of 17.9 ± 1.4, while for the deep extracted DOM, the ratio increased to 30.5 ± 2.3. These differences have been associated with extraction preferences of nonpolar over polar compounds when using PPL resins.^36,37^ For example, it has recently been reported that extracts obtained by SPE with PPL resins seem to be somewhat enriched in N-poor, low molecular weight, and recalcitrant DOM and therefore show less variability than the corresponding bulk DOM.^33^ The implications of this for the description of marine DOM binding remain largely unexplored and will be the subject of future work.

The carbon content of the SPE–DOM extracts was slightly higher for the surface (35.5 ± 0.6%) than for the deep (29.2 ± 0.7%) sample. Marine DOM has been reported to be about 50% C by weight,^31,38^ as estimated for terrigenous humic matter.^39^ We also obtained similar values between 43 ± 1.5 and 53 ± 3.8%, for the mass percentage of C for SPE–DOM samples from the Baltic Sea.^5^ However, at present, we have no explanation as to why the carbon content of our extracted DOM was lower in this case.

### 3.2 North Atlantic DOM Proton Binding Groups

**Figure 1:**
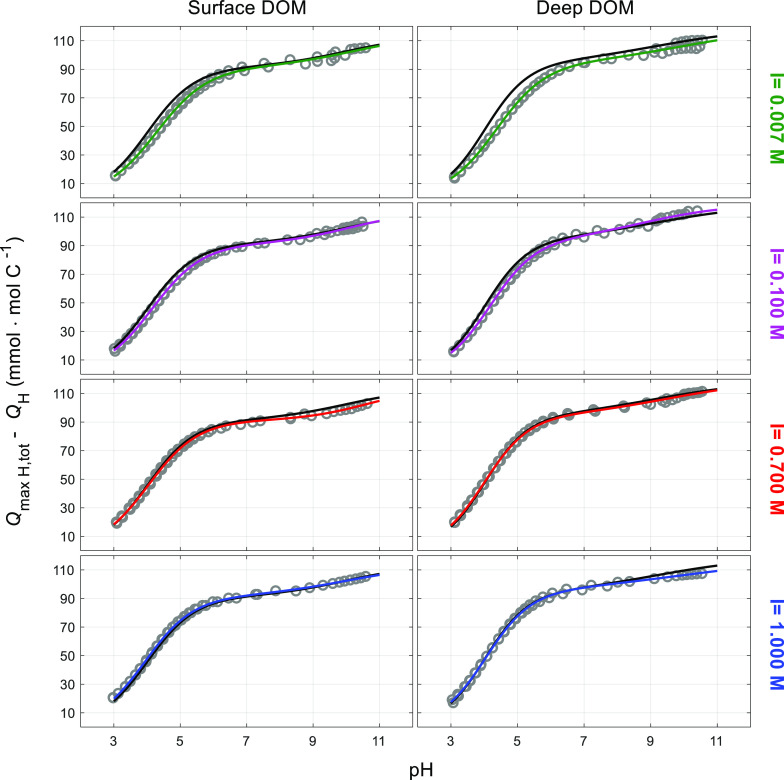
NICA–Donnan fits to proton titration data of North Atlantic SPE–DOM at 25 °C and 0.007, 0.1, 0.7, and 1.0 M ionic strength, using the PB–*V*_D_ model: surface (left panel) and deep (right panel) samples. Symbols: experimental values. Colored lines: model fits at each ionic strength. The uppermost black curve corresponds to the charge “master curve” (*Q*_maxH,tot_ – *Q*_H_) vs pH_D_.

We carried out experimental acid–base titration curves for the surface and deep SPE–DOM at 25 °C at four different ionic strengths. We expressed the DOM charge (*q*_DOM_) as the difference between the total amount of binding sites available for protons and the binding sites already occupied by protons (*q*_DOM_= *Q*_maxH,tot_ – *Q*_H_), i.e., the chemical groups that are free at any point of the titration. Experiments at each ionic strength were done in duplicate, and the combination of those data sets was fitted to the NICA–Donnan model (Figure 1).

The total amount of proton binding groups in the North Atlantic SPE–DOM (*Q*_maxH,tot_) was not significantly different between the surface (112.7 ± 5.8 mmol·mol C^–1^) and deep (117.3 ± 5.6 mmol·mol C^–1^) samples. The total proton binding represents a maximum of binding sites for chemical species that can compete with protons for the DOM binding sites, with a predominantly covalent behavior,^40^ e.g., most trace metals in seawater. On average, 11.4 ± 0.6% of the C atoms in our extracted North Atlantic DOM therefore have a functional group with a binding site for ionic species. Considering the DOC concentrations (84.5 and 47.9 μmol·L^–1^) measured in the surface and deep waters at our tropical North Atlantic sampling sites, the DOM contains about 9.5 and 5.6 μmol·L^–1^ of acid–base groups, respectively.

Even if we restrict our proton binding curves (Figure 1) to the pH window usually used in alkalinity titrations (from pH 8 to 3), our predicted DOM contribution to organic alkalinity, with the observed chemical binding heterogeneity, is between about 4 and 7 μmol·L^–1^ for North Atlantic deep and surface waters, respectively.

Currently, organic alkalinity is thought to be responsible for a “missing” 5 μmol·kg^–1^ contribution to total alkalinity in the open ocean^41^ and may represent a more important but poorly understood component of total alkalinity in coastal waters.^42^

**Figure 2:**
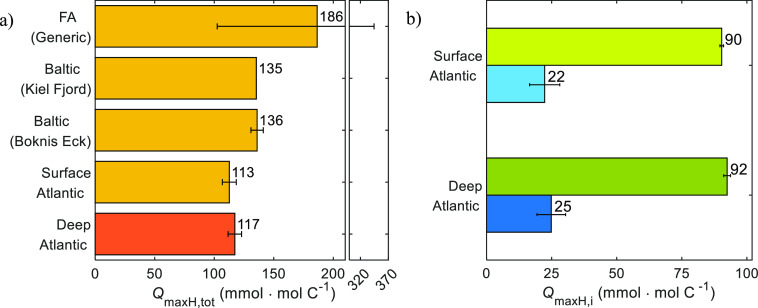
Total amount of proton binding groups obtained from the fits of the NICA–Donnan model (PB–*V*_D_) to the proton binding data shown in Figure 1: *Q*_maxH,tot_ (a) and *Q*_maxH,i_ (b), for the low affinity (DOM_1_, green bars) and high affinity (DOM_2_, blue bars) distributions. The values of Baltic (Boknis Eck) and Baltic (Kiel Fjord) are from Lodeiro et al.^4,5^ Bar heights indicate the mean, and error bars indicate the standard deviation. The FA values indicate *Q*_maxH_ for a generic terrestrial fulvic acid from Milne et al.^16^ (calculated with the standard *V*_D_ model); the error bar indicates the range of values used for the derivation of the generic value.

The obtained total amount of ion binding groups for open ocean DOM is slightly lower than observed for coastal DOM (135–136 mmol·mol C^–1^), and much lower than the average value reported for a generic fulvic acid of terrestrial origin (186 mmol·mol C^–1^)^16^ as shown in Figure 2a, which will have an impact in trace metal biogeochemistry and alkalinity.

The amount of DOM_1_ binding groups (*Q*_maxH,1_) is higher than that of DOM_2_ groups (*Q*_maxH,2_), with a *Q*_maxH,2_/*Q*_maxH,1_ ratio of 0.25 ± 0.07 and 0.27 ± 0.06 for surface and deep samples, respectively (Figure 2b). Our ratios are similar to previous values of 0.27–0.37 observed for coastal DOM and a generic fulvic acid.^4,5,16^ The fraction of the low affinity groups (DOM_1_) in the North Atlantic SPE–DOM remains therefore constant with depth (79–80% of the total binding groups), though other authors observed an increase in open ocean DOM samples.^29^ This discrepancy could be ascribed to the differences in the experimental techniques used (proton binding vs structural analysis).

### 3.3 North Atlantic DOM Intrinsic Proton Binding Parameters

We used the Donnan model to account for the electrostatic contribution to the ion binding by DOM. This model assumes a homogeneous distribution of fixed charges resulting from the dissociation of proton binding functional groups in DOM and considers that DOM behaves as an electroneutral three-dimensional permeable gel that chemical species can penetrate. Experimental measurements of the Donnan volume (*V*_D_) are inaccurate,^43^ so we used two different fitted values for DOM: 1) *V*_D_ consistent with Poisson–Boltzmann (PB–*V*_D_) and 2) standard *V*_D_ model. Our fitting criterion of convergence was the merging of the obtained charge curves at different ionic strengths when they were plotted against the local pH value in the Donnan volume, pH_D_, (−log *c*_H,D_), which is termed the “master curve”.^23^

At high ionic strength, the electrostatic contribution to the ion binding is expected to be negligible, and the master curve should overlap with the charge curve obtained at 1 M ionic strength. Nevertheless, application of the standard *V*_D_ Donnan model resulted in a master curve that deviated significantly from the titration curve obtained at 1 M ionic strength (Figure S3). Application of the PB–*V*_D_ model (Figure 1) resulted in improved overlap between the master curve and the charge curve at 1 M ionic strength, which is reasonable.

**Figure 3:**
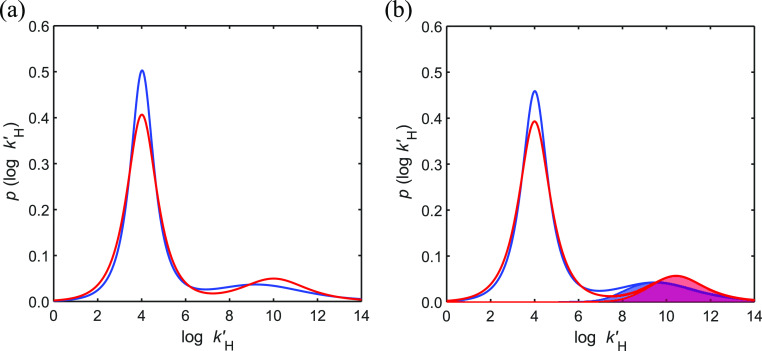
Proton binding affinity spectra of North Atlantic SPE–DOM at 25 °C calculated from the NICA–Donnan model (PB–*V*_D_) parameters of Table S3 for surface (red lines) and deep (blue lines) samples: (a) intrinsic values and (b) effective values calculated at I = 0.7 M and density of protonated sites (occupation of binding sites by protons) at the experimental values of pH 8.08 (shaded red area) and pH 7.56 (shaded blue area) of the surface and deep samples, respectively. All spectra are normalized to one.

Moreover, with the fitted binding parameters reported here, we could calculate for the first time the intrinsic proton binding affinity spectra for open ocean DOM (Figure 3a).

**Figure 4:**
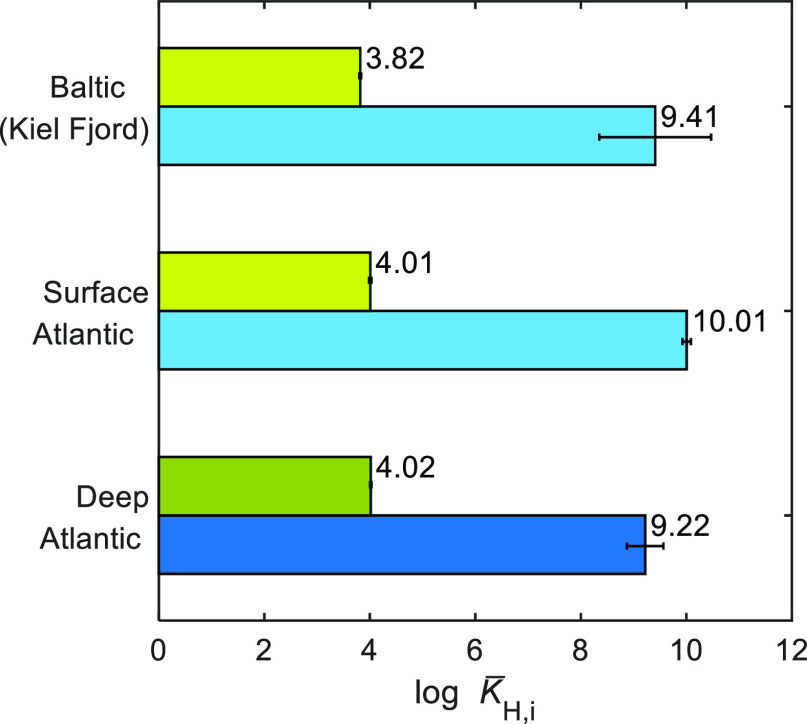
Median values of the intrinsic proton binding affinity (log *K̅*_H,*i*_) parameters obtained from the fits of the NICA–Donnan model (PB–*V*_D_) to proton binding data shown in Figure 1: low affinity (DOM_1_, green bars) and high affinity distribution (DOM_2_, blue bars). Bar heights indicate the mean, and error bars indicate the standard deviation. The values for the Baltic (Kiel Fjord) are from Lodeiro et al.^4^

The affinity spectra represent the density of the probability of proton binding affinity (i.e., the fraction of binding sites with a specific value of the microscopic binding constant log *k*′_H_) and are therefore a key tool to determine the intrinsic characteristics of the binding sites potentially available for chemical species (e.g., trace metals) in the open ocean. The affinity spectra reflect the complex mixture of marine DOM and indicate its chemical reactivity. Therefore, we provide both the proton affinity distributions (Figure 3) and the median value of these distributions (log *K̅*_H*,i*_) for marine DOM (Figure 4).

Surface and deep intrinsic binding spectra showed significant differences, reflecting the changes in log *K̅*_H*,i*_ and *m_i_* values with depth (Figure 3a). For the low affinity group of binding sites (DOM_1_), the values of log *K̅*_H,1_ are very similar for surface (4.01 ± 0.02) and deep (4.02 ± 0.02) samples (Figures 3a and 4). On the other hand, and despite the larger uncertainties, the log *K̅*_H,2_ values for the high affinity groups (DOM_2_) show a clear difference between the surface (10.01 ± 0.08) and deep (9.22 ± 0.35) SPE–DOM samples (Figures 3a and 4). Therefore, the affinity spectra showed an overall shift for the DOM_2_ distribution toward lower affinity values from the surface to depth (Figure 3a).

**Figure 5:**
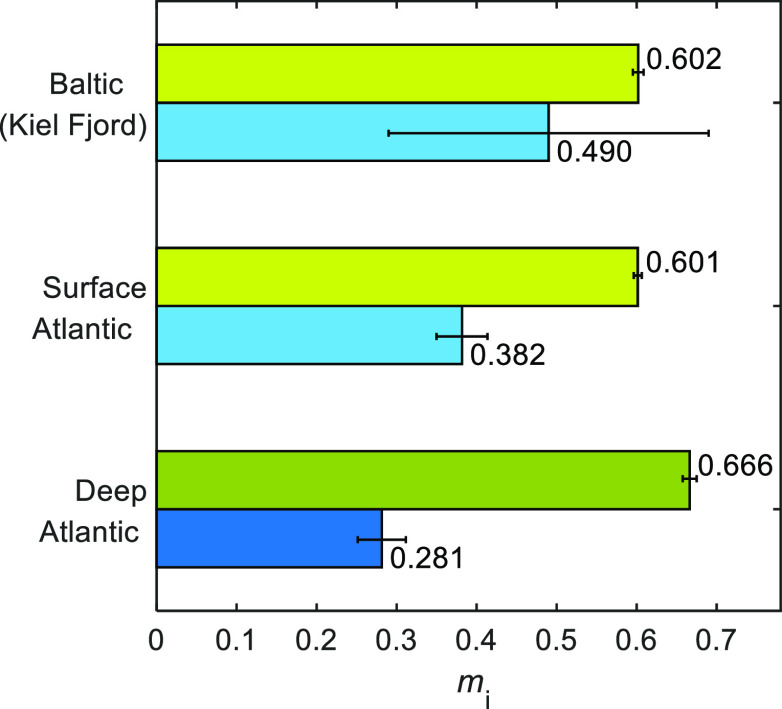
Chemical binding heterogeneity (*m*_*i*_) parameters obtained from the fits of the NICA–Donnan model (*PB*–*V*_D_) to proton binding data shown in Figure 1 for the low affinity (DOM_1_, green bars) and high affinity (DOM_2_, blue bars) distributions. Bar heights indicate the mean, and error bars indicate the standard deviation. The values for the Baltic (Kiel Fjord) are from Lodeiro et al.^4^

The chemical binding heterogeneity of DOM (*m_i_*) is a key parameter not constrained yet for open ocean samples. This parameter is related to the width of the ion affinity distribution function (affinity spectra, Figure 3), which reflects the chemical binding heterogeneity. It can be hypothesized that this heterogeneity parameter could also serve as a proxy for DOM molecular diversity. We calculated an intrinsic *m*_1_ value for North Atlantic SPE–DOM of 0.601 ± 0.005 for the DOM_1_ groups in the surface and of 0.666 ± 0.009 in the deep samples (Figure 5). In contrast, the intrinsic *m*_2_ value of the high affinity DOM_2_ groups in the deep sample (0.28 ± 0.03) was lower (i.e., more heterogeneous) than that observed in the surface sample (0.38 ± 0.03). This agrees with the wider affinity distribution function for DOM_1_ groups and the narrower distribution for the DOM_2_ group of the surface compared to the deep sample (Figure 3a).

Current intrinsic and emergent recalcitrant concepts of marine DOM persistence^44^ are reflected in the obtained heterogeneity values. The greater heterogeneity of the DOM_2_ compared to the DOM_1_ group distribution in open ocean SPE–DOM indicates that the low affinity binding sites behaved closer to what would be expected for a simple carboxylic chemical group. On the contrary, the high heterogeneity (low *m*_2_ values) of DOM_2_ probably reflects the characteristics of aromatic carbon compounds (e.g., phenols), which are difficult to degrade and thus accumulate in a larger variety of chemical species of lower abundance than the easier degradable/mineralized DOM_1_ groups that accumulate in the deep ocean as more homogeneous chemical compounds.

We compared our open ocean data with the only available intrinsic proton binding parameters for a coastal DOM^4^ (Figures 4 and 5). Both data sets were obtained with the NICA-Donnan model, using an expression for *V*_D_ consistent with the NLPB equation (PB-*V*_D_). The DOM_1_ group of the North Atlantic DOM presents log *K̅*_H,1_ values higher than those observed in coastal waters (3.82 ± 0.02). This DOM_1_ distribution of our deep open ocean DOM is ca. 10% more homogeneous than the surface sample, which is very similar to that previously reported for a coastal surface DOM (Figure 5). Direct comparison of the open ocean and coastal DOM high affinity groups for protons is not possible, since the intrinsic data of DOM_2_ for the Baltic Sea (Kiel Fjord) were estimated at I = 0.7 M using the generic intrinsic NICA parameters for fulvic acid.

Intrinsic binding affinities depend on the electrostatic model, and therefore comparison of NICA-Donnan constants derived using the PB-*V*_D_ and standard *V*_D_ models is not straightforward. The Donnan model with the PB*-V*_D_ equation has not been used so far to account for the electrostatic contribution to the binding by DOM in natural waters; hence, the comparison of our intrinsic ion binding affinity values with, e.g., generic fulvic or humic acids of terrestrial origin or effective ion binding constants for marine humic substances is not possible. Therefore, to compare intrinsic binding data for terrestrial and marine DOM, we included the data fitted to a standard *V*_D_ Donnan model in the Supporting Information. Nevertheless, the use of the PB-*V*_D_ model is recommended over that of the standard *V*_D_ expression. Among the advantages of the former, we highlight its direct connection with the NLPB treatment for different geometries, its independency of the kind of macromolecular ligand (e.g., DOM), homogeneous or heterogeneous, and the explicit consideration of the DOM charge in the calculation of *V*_D_.^24^

### 3.4 North Atlantic DOM Effective Proton Binding Parameters at 0.7 M Ionic Strength

**Figure 6:**
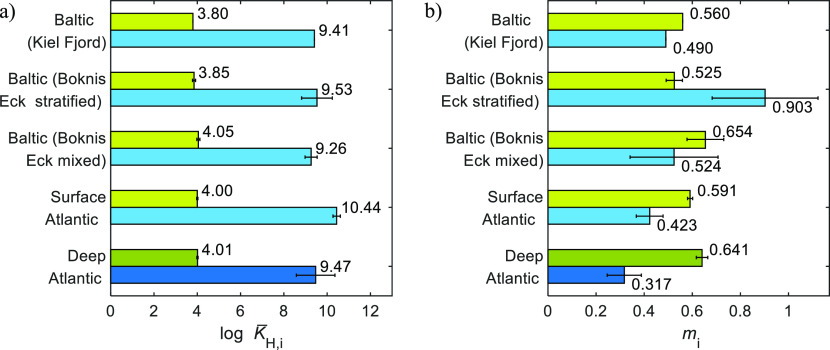
Effective binding parameters estimated at I = 0.7 M, using the intrinsic NICA-Donnan model with PB-*V*_D_, and the proton binding data shown in Figure 1 for the DOM_1_ (green bars) and DOM_2_ (blue bars) distributions: log *K̅*_H,*i*_ (a) and *m*_*i*_ (b). The values Baltic (Boknis Eck) and Baltic (Kiel Fjord) are from Lodeiro et al.^4,5^ Bar heights indicate the mean, and error bars indicate the standard deviation.

In addition to the DOM intrinsic proton binding affinities, we also calculate the effective binding parameters at I = 0.7 M for the sampling site in the North Atlantic (Figure 6, Table S3). The effective parameters combine both the chemical and electrostatic contributions to the binding. We anticipate a minor electrostatic input to the ion binding at the high ionic strength (ca. 0.7 M) of open ocean waters. Therefore, the calculated effective DOM binding parameters at I = 0.7 M are very similar to the intrinsic ones (Table S3).

Surface and deep samples presented similar values for the effective proton binding affinity of DOM_1_ groups, although significant differences were observed for the high affinity group values. The marine DOM_1_ group at 0.7 M ionic strength had slightly higher effective binding affinities than those previously calculated for stratified coastal DOM at the same ionic strength and at 0.32 M (salinity 16, Kiel Baltic Fjord), while for the DOM_2_ groups, the increase was only observed for the marine surface sample (Figure 6a).

Moreover, the effective *m* values at I = 0.7 M for DOM_1_ were lower than the calculated intrinsic ones (Table S3). Regarding surface and deep samples, we observed the same tendency for the effective values with *m*_1_ increasing and *m*_2_ decreasing with depth (Figure 6b). Therefore, the DOM_1_ groups of marine DOM became less heterogeneous with depth, while the opposite was observed for the DOM_2_ groups.

Compared to coastal DOM, the effective *m*_1_ value calculated at 0.7 M ionic strength for the deep Atlantic sample is similar to a Baltic mixed but higher than a stratified sample as described in Lodeiro et al.^5^ The surface Atlantic DOM has an effective *m*_1_ value between that observed for coastal and deep marine DOM (Figure 6b). On the contrary, the DOM_2_ groups of North Atlantic SPE–DOM are markedly more heterogeneous than coastal SPE–DOM at I = 0.7 M.

We also calculated the distribution of sites occupied by protons from the effective affinity spectrum of marine DOM (I = 0.7 M) at the measured pH values of surface (8.08) and deep (7.56) seawater samples (Figure 3b). These distributions reflect the affinity of the sites actually involved in proton exchange under the relevant environmental conditions. It is straightforward to prove that at a given pH value the sites with proton affinity log *k*′_H_ = pH are half occupied. Note that in both cases (surface and deep samples) the DOM_2_ groups are mostly protonated at the experimental pH, compared to the DOM_1_ sites, which are essentially unoccupied. This is a result of the relative values of pH and the central values (log *K̅*_H,*i*_ values) and widths (*m*_*i*_) of each mode of groups.^22^ Consequently, it is expected that the DOM_1_ distribution will have little relevance to proton competition with trace metal ions for binding by DOM in marine environments. When comparing the fractional occupation of sites between surface and deep samples, we observe that, in the latter case, the density distribution of protonated sites became broader and shifts toward lower affinity values. This is a result of the lower pH values (7.56 vs 8.08), log *K̅*_H,2_ (9.5 vs 10.4), and *m*_2_ (0.32 vs 0.42) of deep DOM compared to surface DOM. This might indicate that metal-proton exchange in marine DOM is more relevant for aged organic matter.

Our data show that the average affinity and binding heterogeneity of the DOM_2_ groups could underpin the chemical reactivity of marine DOM and shed some light on explaining its stabilization and persistence in seawater. Moreover, as we also determined intrinsic parameters, it would be possible to extrapolate beyond the sample conditions, though the electrostatic model used is critical for the binding description and a correct extrapolation.^24^

Nevertheless, the information obtained from the binding affinity spectra of protons does not provide a straightforward perception of how the ion–DOM binding behavior will be in natural conditions, and an evolution of the spectra from the monocomponent system calculated here to the multicomponent seawater mixture can be expected.^22^

Further efforts are needed to solve the current lack of knowledge on whether and how the thermodynamic properties of marine DOM affect the interactions with major seawater components (e.g., Ca and Mg) and trace metals. For example, the determination of the conditional affinity spectrum (CAS) for the ionic species present in seawater under a given constraint (pH, concentration of competing ions, etc.) will enable us to determine the effect of all interfering cations on the binding affinity distribution of a given one. The study of the CAS seen by a trace metal ion binding to marine DOM can allow the assessment of its effective binding affinity and heterogeneity as a function of the concentration of competing species (e.g., H^+^).^45^ We hypothesize that, at the natural pH of seawater, the influence of proton ions is probably the most important contribution for ions competing for the high affinity sites of DOM. In addition to the pH, temperature and pressure effects on metal binding by marine DOM also need to be constrained to study the implications of expected changes under ongoing climate change.

## Data Availability

The data sets generated and/or analyzed during the current study are available in the attached spreadsheet.

## Supporting Information Available

1. Sampling map (Figure S1, page S2); 2. Seawater analysis (page S3): North Atlantic seawater main characteristics (Table S1) and North Atlantic seawater micro- and macronutrient concentrations (Table S2); 3. Strategy for the derivation of NICA–Donnan (PB–*V*_D_) model parameters (page S4); 4. Intrinsic and effective proton binding parameters calculated using the NICA–Donnan (PB–*V*_D_) model (pages S5–S6): Optimized NICA–Donnan (PB–*V*_D_) parameter values (Table S3), NICA–Donnan (PB–*V*_D_) fits (Figure S2); 5. Potentiometric titration data fitted to a standard *V*_D_ Donnan model (pages S7–S10): NICA-Donnan fits (Figures S3), proton binding affinity (log *K̅*_H,*i*_) (Figure S4) and heterogeneity (*m*_*i*_) parameters (Figure S5); 6. Glossary of terms (pages S11–S12) (PDF)
Complete experimental data set (XLSX)

## References

[ref1] Hansell D.; Carlson C.; Repeta D.; Schlitzer R. Dissolved Organic Matter in the Ocean: A Controversy Stimulates New Insights. Oceanography 2009, 22 (4), 202–211. 10.5670/oceanog.2009.109.

[ref2] Carlson C. A.; Hansell D. A. DOM Sources, Sinks, Reactivity, and Budgets. In Biogeochemistry of Marine Dissolved Organic Matter; Hansell D. A., Carlson C. A., Eds.; Elsevier: Boston, 2015; pp 65–126, 10.1016/B978-0-12-405940-5.00003-0.

[ref3] Zhu K.; Hopwood M. J.; Groenenberg J. E.; Engel A.; Achterberg E. P.; Gledhill M. Influence of pH and Dissolved Organic Matter on Iron Speciation and Apparent Iron Solubility in the Peruvian Shelf and Slope Region. Environ. Sci. Technol. 2021, 55 (13), 9372–9383. 10.1021/acs.est.1c02477. 34110803

[ref4] Lodeiro P.; Rey-Castro C.; David C.; Achterberg E. P.; Puy J.; Gledhill M. Acid-Base Properties of Dissolved Organic Matter Extracted from the Marine Environment. Sci. Total Environ. 2020, 729, 138437 10.1016/j.scitotenv.2020.138437. 32371203

[ref5] Lodeiro P.; Rey-Castro C.; David C.; Puy J.; Achterberg E. P.; Gledhill M. Seasonal Variations in Proton Binding Characteristics of Dissolved Organic Matter Isolated from the Southwest Baltic Sea. Environ. Sci. Technol. 2021, 55 (23), 16215–16223. 10.1021/acs.est.1c04773. 34766769 PMC8719755

[ref6] Middelburg J. J.; Soetaert K.; Hagens M. Ocean Alkalinity, Buffering and Biogeochemical Processes. Rev. Geophys. 2020, 58 (3), e2019RG000681 10.1029/2019RG000681. PMC7391262 32879922

[ref7] Seidel M.; Vemulapalli S. P. B.; Mathieu D.; Dittmar T. Marine Dissolved Organic Matter Shares Thousands of Molecular Formulae Yet Differs Structurally across Major Water Masses. Environ. Sci. Technol. 2022, 56 (6), 3758–3769. 10.1021/acs.est.1c04566. 35213127

[ref8] Zark M.; Christoffers J.; Dittmar T. Molecular Properties of Deep-Sea Dissolved Organic Matter Are Predictable by the Central Limit Theorem: Evidence from Tandem FT-ICR-MS. Mar. Chem. 2017, 191, 9–15. 10.1016/j.marchem.2017.02.005.

[ref9] Gledhill M.; Gerringa L. J. A. The Effect of Metal Concentration on the Parameters Derived from Complexometric Titrations of Trace Elements in Seawater—A Model Study. Front. Mar. Sci. 2017, 4, 1–15. 10.3389/fmars.2017.00254.

[ref10] Koopal L.; Tan W.; Avena M. Equilibrium Mono- and Multicomponent Adsorption Models: From Homogeneous Ideal to Heterogeneous Non-Ideal Binding. Adv. Colloid Interface Sci. 2020, 280, 102138 10.1016/j.cis.2020.102138. 32387754

[ref11] Catalá T. S.; Shorte S.; Dittmar T. Marine Dissolved Organic Matter: A Vast and Unexplored Molecular Space. Appl. Microbiol. Biotechnol. 2021, 105 (19), 7225–7239. 10.1007/s00253-021-11489-3. 34536106 PMC8494709

[ref12] Kerr D. E.; Brown P. J.; Grey A.; Kelleher B. P. The Influence of Organic Alkalinity on the Carbonate System in Coastal Waters. Mar. Chem. 2021, 237, 104050 10.1016/j.marchem.2021.104050.

[ref13] Sharp J. D.; Byrne R. H. Interpreting Measurements of Total Alkalinity in Marine and Estuarine Waters in the Presence of Proton-Binding Organic Matter. Deep-Sea Res. Part Oceanogr. Res. Pap. 2020, 165, 103338 10.1016/j.dsr.2020.103338.

[ref14] Ardiningsih I.; Krisch S.; Lodeiro P.; Reichart G.-J.; Achterberg E. P.; Gledhill M.; Middag R.; Gerringa L. J. A. A. Natural Fe-Binding Organic Ligands in Fram Strait and over the Northeast Greenland Shelf. Mar. Chem. 2020, 224, 103815 10.1016/j.marchem.2020.103815.

[ref15] Ye Y.; Völker C.; Gledhill M. Exploring the Iron-Binding Potential of the Ocean Using a Combined pH and DOC Parameterization. Glob. Biogeochem. Cycles 2020, 34 (6), 1–16. 10.1029/2019GB006425.

[ref16] Milne C. J.; Kinniburgh D. G.; Tipping E. Generic NICA-Donnan Model Parameters for Proton Binding by Humic Substances. Environ. Sci. Technol. 2001, 35 (10), 2049–2059. 10.1021/es000123j. 11393987

[ref17] Hollister A. P.; Whitby H.; Seidel M.; Lodeiro P.; Gledhill M.; Koschinsky A. Dissolved Concentrations and Organic Speciation of Copper in the Amazon River Estuary and Mixing Plume. Mar. Chem. 2021, 234 (June), 104005 10.1016/j.marchem.2021.104005.

[ref18] Millero F. J. The Physical Chemistry of Natural Waters; Wiley - Interscience Series in Geochemistry: 2000.

[ref19] Koopal L. K.; van Riemsdijk W. H.; de Wit J. C. M.; Benedetti M. F. Analytical Isotherm Equations for Multicomponent Adsorption to Heterogeneous Surfaces. J. Colloid Interface Sci. 1994, 166 (1), 51–60. 10.1006/jcis.1994.1270.

[ref20] Kinniburgh D. G.; Milne C. J.; Benedetti M. F.; Pinheiro J. P.; Filius J.; Koopal L. K.; Van Riemsdijk W. H. Metal Ion Binding by Humic Acid: Application of the NICA-Donnan Model. Environ. Sci. Technol. 1996, 30 (5), 1687–1698. 10.1021/es950695h.

[ref21] Kinniburgh D. G.; van Riemsdijk W. H.; Koopal L. K.; Borkovec M.; Benedetti M. F.; Avena M. J. Ion Binding to Natural Organic Matter: Competition, Heterogeneity, Stoichiometry and Thermodynamic Consistency. Colloids Surf. Physicochem. Eng. Asp. 1999, 151 (1–2), 147–166. 10.1016/S0927-7757(98)00637-2.

[ref22] Rey-Castro C.; Mongin S.; Huidobro C.; David C.; Salvador J.; Garcés J. L.; Galceran J.; Mas F.; Puy J. Effective Affinity Distribution for the Binding of Metal Ions to a Generic Fulvic Acid in Natural Waters. Environ. Sci. Technol. 2009, 43 (19), 7184–7191. 10.1021/es803006p. 19848120

[ref23] Benedetti M. F.; vanRiemsdik W. H.; Koopal L. K. Humic Substances Considered as a Heterogeneous Donnan Gel Phase. Environ. Sci. Technol. 1996, 30 (6), 1805–1813. 10.1021/es950012y.

[ref24] Companys E.; Garces J. L.; Salvador J.; Galceran J.; Puy J.; Mas F. Electrostatic and Specific Binding to Macromolecular Ligands - A General Analytical Expression for the Donnan Volume. Colloids Surf. -Physicochem. Eng. Asp. 2007, 306 (1–3), 2–13. 10.1016/j.colsurfa.2007.01.016.

[ref25] Pinheiro J. P.; Rotureau E.; Duval J. F. L. Addressing the Electrostatic Component of Protons Binding to Aquatic Nanoparticles beyond the Non-Ideal Competitive Adsorption (NICA)-Donnan Level: Theory and Application to Analysis of Proton Titration Data for Humic Matter. J. Colloid Interface Sci. 2021, 583, 642–651. 10.1016/j.jcis.2020.09.059. 33039861

[ref26] Wagner S.; Brandes J.; Spencer R. G. M.; Ma K.; Rosengard S. Z.; Moura J. M. S.; Stubbins A. Isotopic Composition of Oceanic Dissolved Black Carbon Reveals Non-Riverine Source. Nat. Commun. 2019, 10 (1), 5064 10.1038/s41467-019-13111-7. 31699996 PMC6838092

[ref27] Li Y.; Harir M.; Lucio M.; Kanawati B.; Smirnov K.; Flerus R.; Koch B. P.; Schmitt-Kopplin P.; Hertkorn N. Proposed Guidelines for Solid Phase Extraction of Suwannee River Dissolved Organic Matter. Anal. Chem. 2016, 88 (13), 6680–6688. 10.1021/acs.analchem.5b04501. 27176119

[ref29] Hertkorn N.; Harir M.; Koch B. P.; Michalke B.; Schmitt-Kopplin P. High-Field NMR Spectroscopy and FTICR Mass Spectrometry: Powerful Discovery Tools for the Molecular Level Characterization of Marine Dissolved Organic Matter. Biogeosciences 2013, 10 (3), 1583–1624. 10.5194/bg-10-1583-2013.

[ref30] Martínez-Pérez A. M.; Osterholz H.; Nieto-Cid M.; Álvarez M.; Dittmar T.; Álvarez-Salgado X. A. Molecular Composition of Dissolved Organic Matter in the Mediterranean Sea. Limnol. Oceanogr. 2017, 62 (6), 2699–2712. 10.1002/lno.10600.

[ref31] Broek T. A. B.; Walker B. D.; Guilderson T. P.; McCarthy M. D. Coupled Ultrafiltration and Solid Phase Extraction Approach for the Targeted Study of Semi-Labile High Molecular Weight and Refractory Low Molecular Weight Dissolved Organic Matter. Mar. Chem. 2017, 194, 146–157. 10.1016/j.marchem.2017.06.007.

[ref32] Bercovici S. K.; Koch B. P.; Lechtenfeld O. J.; McCallister S. L.; Schmitt-Kopplin P.; Hansell D. A. Aging and Molecular Changes of Dissolved Organic Matter Between Two Deep Oceanic End-Members. Glob. Biogeochem. Cycles 2018, 32 (10), 1449–1456. 10.1029/2017GB005854.

[ref33] Jerusalén-Lleó E.; Nieto-Cid M.; Fuentes-Santos I.; Dittmar T.; Álvarez-Salgado X. A. Solid Phase Extraction of Ocean Dissolved Organic Matter with PPL Cartridges: Efficiency and Selectivity. Front. Mar. Sci. 2023, 10, 1159762 10.3389/fmars.2023.1159762.

[ref34] Chaichana S.; Jickells T.; Johnson M. Interannual Variability in the Summer Dissolved Organic Matter Inventory of the North Sea: Implications for the Continental Shelf Pump. Biogeosciences 2019, 16 (5), 1073–1096. 10.5194/bg-16-1073-2019.

[ref35] Letscher R. T.; Moore J. K. Preferential Remineralization of Dissolved Organic Phosphorus and Non-Redfield DOM Dynamics in the Global Ocean: Impacts on Marine Productivity, Nitrogen Fixation, and Carbon Export. Glob. Biogeochem. Cycles 2015, 29 (3), 325–340. 10.1002/2014GB004904.

[ref36] Dittmar T.; Koch B.; Hertkorn N.; Kattner G. A Simple and Efficient Method for the Solid-Phase Extraction of Dissolved Organic Matter (SPE-DOM) from Seawater. Limnol. Oceanogr. Methods 2008, 6 (6), 230–235. 10.4319/lom.2008.6.230.

[ref37] Ksionzek K. B.; Zhang J.; Ludwichowski K.-U.; Wilhelms-Dick D.; Trimborn S.; Jendrossek T.; Kattner G.; Koch B. P. Stoichiometry, Polarity, and Organometallics in Solid-Phase Extracted Dissolved Organic Matter of the Elbe-Weser Estuary. PLoS One 2018, 13 (9), e0203260 10.1371/journal.pone.0203260. 30183724 PMC6124745

[ref38] Green N. W.; Perdue E. M.; Aiken G. R.; Butler K. D.; Chen H.; Dittmar T.; Niggemann J.; Stubbins A. An Intercomparison of Three Methods for the Large-Scale Isolation of Oceanic Dissolved Organic Matter. Mar. Chem. 2014, 161, 14–19. 10.1016/j.marchem.2014.01.012.

[ref39] Tipping E. Cation Binding by Humic Substances; Cambridge University Press: Cambridge, 2002; 10.1017/CBO9780511535598.

[ref40] Lodeiro P.; Martínez-Cabanas M.; Herrero R.; Barriada J. L.; Vilariño T.; Rodríguez-Barro P.; Sastre de Vicente M. E. The Proton Binding Properties of Biosorbents. Environ. Chem. Lett. 2019, 17 (3), 1281–1298. 10.1007/s10311-019-00883-z.

[ref41] Fong M. B.; Dickson A. G. Insights from GO-SHIP Hydrography Data into the Thermodynamic Consistency of CO2 System Measurements in Seawater. Mar. Chem. 2019, 211, 52–63. 10.1016/j.marchem.2019.03.006.

[ref42] Kerr D. E.; Turner C.; Keogh J.; Grey A.; Brown P. J.; Kelleher B. P. OrgAlkCalc: Estimation of Organic Alkalinity Quantities and Acid-Base Properties with Proof of Concept in Dublin Bay. Mar. Chem. 2023, 251, 104234 10.1016/j.marchem.2023.104234.

[ref43] Avena M. J.; Vermeer A. W. P.; Koopal L. K. Volume and Structure of Humic Acids Studied by Viscometry pH and Electrolyte Concentration Effects. Colloids Surf., A 1999, 151, 213–224. 10.1016/S0927-7757(98)00504-4.

[ref44] Dittmar T.; Lennartz S. T.; Buck-Wiese H.; Hansell D. A.; Santinelli C.; Vanni C.; Blasius B.; Hehemann J. H. Enigmatic Persistence of Dissolved Organic Matter in the Ocean. Nat. Rev. Earth Environ. 2021, 2 (8), 570–583. 10.1038/s43017-021-00183-7.

[ref45] David C.; Mongin S.; Rey-Castro C.; Galceran J.; Companys E.; Garcés J. L.; Salvador J.; Puy J.; Cecilia J.; Lodeiro P.; Mas F. Competition Effects in Cation Binding to Humic Acid: Conditional Affinity Spectra for Fixed Total Metal Concentration Conditions. Geochim. Cosmochim. Acta 2010, 74 (18), 5216–5227. 10.1016/j.gca.2010.06.023.

