# Supplementary material for: Proton Binding Characteristics of Dissolved Organic Matter Extracted from the North Atlantic
Source: Environ Sci Technol. 2023 Dec 5;57(50):21136–44. doi: 10.1021/acs.est.3c01810 (PMC10734258; doi:10.1021/acs.est.3c01810)
Supplement: Supplementary file 1 — es3c01810_si_001.pdf [file es3c01810_si_001.pdf]

Supporting information

Proton binding characteristics of dissolved organic matter extracted from the North Atlantic

Pablo Lodeiro[a*], Carlos Rey-Castro[a], Calin David[a], Matthew P. Humphreys[b] and Martha Gledhill[c]

[a]Department of Chemistry, Physics, Environmental and Soil sciences, University of Lleida – AGROTECNIO-CERCA Center, Rovira Roure 191, 25198, Lleida, Spain.

[b]NIOZ Royal Netherlands Institute for Sea Research, Department of Ocean Systems (OCS), PO Box 59, 1790 AB Den Burg (Texel), the Netherlands.

[c]GEOMAR Helmholtz Centre for Ocean Research Kiel, Wischhofstraße 1-3, 24148 Kiel, Germany.

*Corresponding author: pablo.lodeiro@udl.cat

1. Sampling map (page S2)

2. Seawater analysis (page S3)

3. Strategy for the derivation of NICA–Donnan model parameters (page S4)

4. Intrinsic and effective proton binding parameters calculated using a NICA-Donnan model with V_D consistent with the non-linear Poissson-Boltzman equation (PB-V_D) (pages S5-S6)

5. Potentiometric titration data fitted to a standard V_D Donnan model (pages S7-S10)

6. Glossary of terms (pages S11-S12)

References (pages S13-S14)

14 pages, 5 figures, 3 tables and a glossary of terms

1. Sampling map

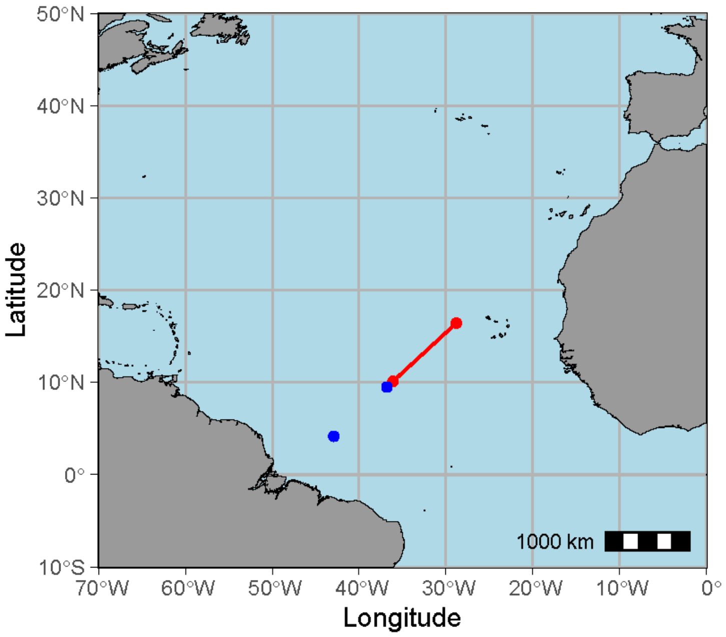

Figure S1. Sampling map. The line that joins the two red dots represent the area where the surface sample was collected whilst the ship was underway. The blue points are the two locations where the deep water samples (ca. 500 m) were collected.

2. Seawater analysis

Table S1. North Atlantic seawater main characteristics (temperature, salinity, pH and oxygen) dissolved organic carbon (DOC) and total dissolved nitrogen (TDN) at surface (ca. 2 m) and depth (500 m).

Sample	DOC $\mu mol \cdot L^{-1}$	TDN $\mu mol \cdot L^{-1}$	T ºC	Salinity	pH	O_2 $mg \cdot L^{-1}$
Surface	84.5 ± 4.4	6.5 ± 0.5	26.3*	36.40*	8.08*	206*
Deep 1	47.9 ± 5.3	37.4 ± 3.7	8.60	34.90	7.790 – 7.556**	1.14
Deep 2			6.49	34.60	7.807 – 7.571***	1.94

*Data from Glodapv2.2021[1]. Accession number 19248, 29°W, 16°N. Cruise - CLIVAR A16N_2003. Station 100, Depth 3 m. DOC concentration of 74.3 $\mu mol \cdot kg^{-1}$
**Data from Glodap v2.2021[1]. Accession number 20982, depth 500 m, position 35°W/7.001°N, Salinity 34.682, temperature 7.07 °C
***Data from Glodapv2.2021[1]. Accession number 18162, depth 500 m, position 40.9°W/5.3°N, Salinity 34.647, temperature 7.04 °C

Table S2. North Atlantic seawater micro- and macronutrient concentrations.

Sample	Al $nmol \cdot L^{-1}$	Fe $\mu mol \cdot L^{-1}$	NO_3^- $\mu mol \cdot L^{-1}$	NO_2^- $\mu mol \cdot L^{-1}$	PO_4^{-3} $\mu mol \cdot L^{-1}$	SiO_4 $\mu mol \cdot L^{-1}$
Surface	24*	0.4**	0.08	0	0	0.7
Deep 1	7.08***	0.99***	35.1±2.5	0.011±0.05	2.17±0.15	16.6±1.9
Deep 2			34.2±2.1	0	2.22±0.13	21.1±1.5

*D361, GA03. FISH sample 222. 15.8N, 28.7W[2].
**Fe data[3] is from flow injection analysis (FIA) not ICP-MS.
***Data from Geotraces GA02 at 500 m from station 37-39[4].

3. Strategy for the derivation of NICA–Donnan model parameters

The experimental datasets of titrant volume and pH (free scale) were converted into pH and charge curves using mass and charge balance relationships, as detailed in the supporting information of Lodeiro *et al.*[5]. The optimization of the NICA–Donnan parameters was carried out by non–linear regression using MATLAB to minimize the root–mean square error (RMSE, in mol·kg DOM^{-1}) in the DOM charge:

$$\text{RMSE}=\left[\frac{\sum_{i=1}^{N}(Q_i-\hat{Q}_i)^2}{N-l}\right]^{1/2} \qquad (1S)$$

where Q_i and $\hat{Q}_i$ are the experimental and fitted values of DOM charge, respectively; N is the number of data points and l is the number of model parameters. We used the MATLAB function “fminsearchbnd”, which finds the minimum of a bound constrained multivariable function using derivative–free method. Q values in mol·kg DOM^{-1} were subsequently transformed to mmol·mol C^{-1} using the calculated carbon content of the DOM extracts. The number of DOM binding sites is not affected by electrostatic effects, and so we considered that the ratio $Q_{\mathrm{maxH},2}$/ $Q_{\mathrm{maxH},1}$ remains constant when calculated the specific binding parameters.

4. Intrinsic and effective proton binding parameters calculated using a NICA-Donnan model with V_D consistent with the non-linear Poissson-Boltzman equation (PB-V_D) and fits to proton titration data.

Table S3. Optimized NICA−Donnan (PB−V_D) parameter values for proton binding at 25ºC. $Q_{maxH,j}$: total amount of proton binding sites within each distribution; log $\overline{K}_{H,j}$: median value of the jth affinity distribution for protons (the proton binding affinity); m_j: chemical binding heterogeneity; a: fitted parameter of the V_D equation[6]: $V_D = \frac{1}{4a\sqrt{I+a^2Q^2z^2}}$, where I is the ionic strength, Q is the surface charge density and z de valence of the symmetric background electrolyte; RMSE: root−mean square error.

	Intrinsic parameters Surface	Intrinsic parameters Deep	Effective parameters* Surface	Effective parameters* Deep
$Q_{maxH,1}$ (mmol·mol C^{-1})	90.3 ± 0.7	92 ± 1	90.3 ± 0.7	92 ± 1
log $\overline{K}_{H,1}$	4.01 ± 0.02	4.02 ± 0.02	4.00 ± 0.03	4.01 ± 0.03
m_1	0.601 ± 0.005	0.666 ± 0.009	0.59 ± 0.01	0.64 ± 0.02
$Q_{maxH,2}$ (mmol·mol C^{-1})	22 ± 6	25 ± 5	22 ± 6	25 ± 5
log $\overline{K}_{H,2}$	10.01 ± 0.08	9.2 ± 0.4	10.4 ± 0.2	9.5 ± 0.9
m_2	0.38 ± 0.03	0.28 ± 0.03	0.42 ± 0.06	0.32 ± 0.07
Fitted parameter *a*	0.017	0.028	−	−
RMSE (mmol·mol C^{-1})	0.893	1.01	0.771	0.718

*Estimated at I = 0.7 M using parameters from the intrinsic NICA-Donnan with PB-V_D.

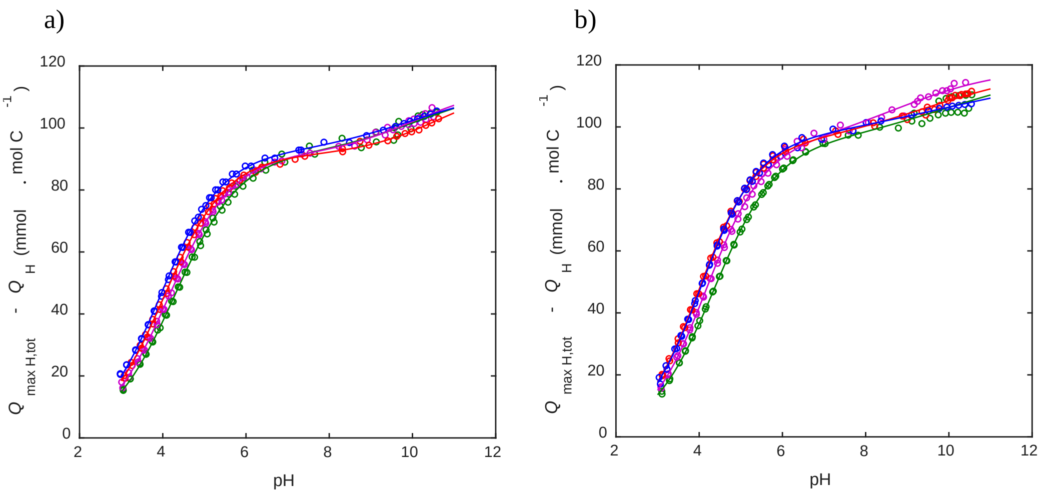

Figure S2. NICA−Donnan fits to proton titration data of North Atlantic SPE−DOM at 25 °C using PB−V_D model: surface (a) and deep (b) samples. Symbols: experimental values. Colored lines correspond to the model fits at each ionic strength: 0.007 (green), 0.1 (pink), 0.7 (red) and 1.0 M (blue).

5. Potentiometric titration data fitted to a standard V_D Donnan model

Experimental acid−base titration curves for the surface and deep North Atlantic DOM were also fitted to the NICA−Donnan model using a standard V_D equation (Figure S3).

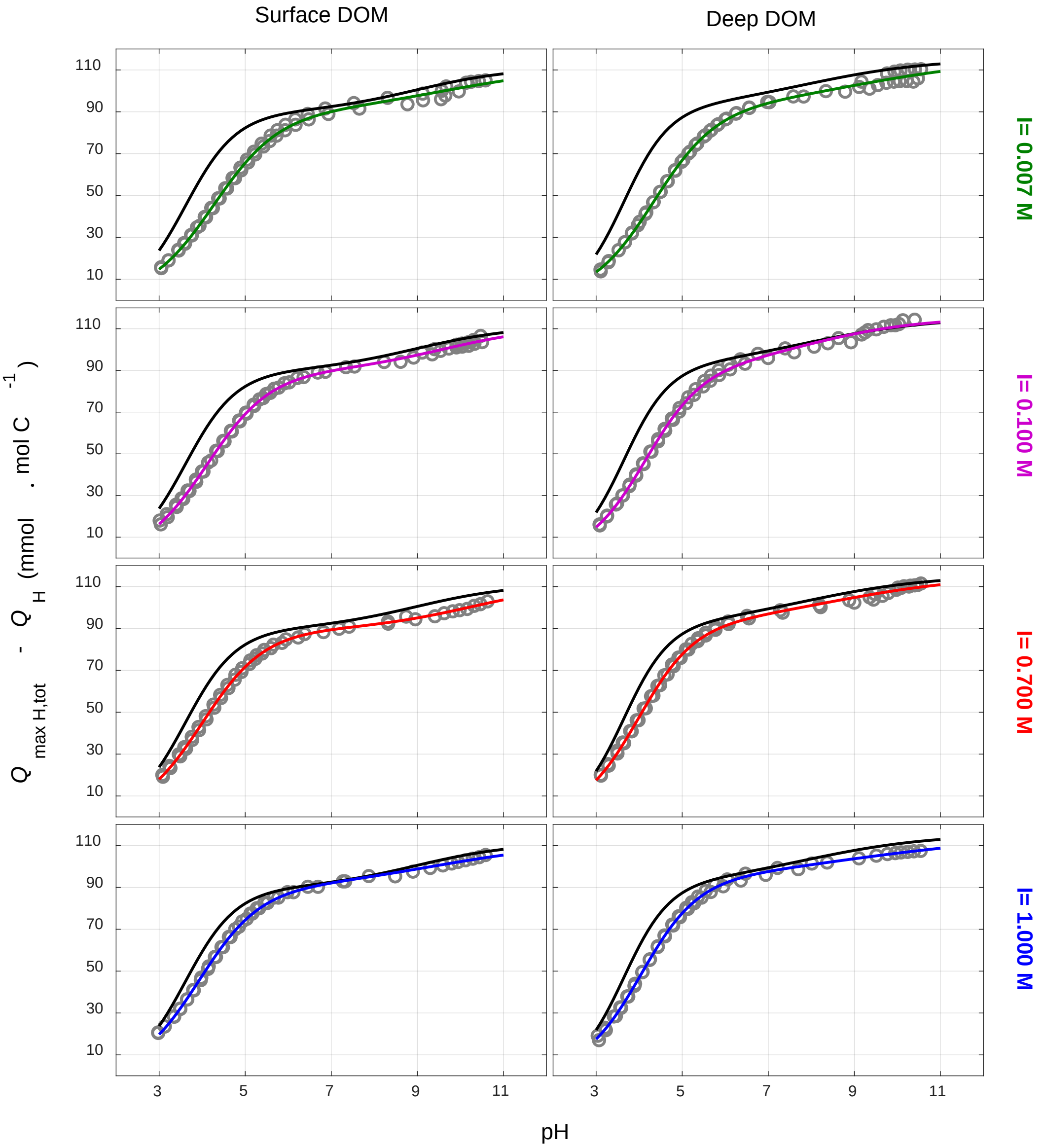

Figure S3. NICA−Donnan fits to proton titration data of North Atlantic SPE−DOM at 25 °C, and 0.007, 0.1, 0.7 and 1.0 M ionic strength, using the standard V_D Donnan model: surface (left panel) and deep samples (right panel). Symbols: experimental values. Colored lines correspond to the model fits at each ionic strength. The uppermost black curve corresponds to the 'master curve' charge ($Q_{maxH,tot}$ - Q_H) *vs*. pH_D.

We calculated median values of the intrinsic proton binding affinity for the DOM_1 mode ($\log \overline{K}_{H,1}$) that are larger than the previously reported for coastal and terrigenous DOM (Figure S3). The values for surface (3.61 ± 0.03) and deep (3.64 ± 0.01) samples were similar. On the other hand, and despite the larger uncertainties obtained, the median values for the intrinsic proton binding affinities for the DOM_2 groups ($\log \overline{K}_{H,2}$) showed a clear difference between the surface (9.0 ± 0.3) and deep (7.9 ± 0.4) samples (Figure S3). Both $\log \overline{K}_{H,2}$values were close to the average reported for a coastal sample (8.6) and a generic fulvic acid (8.6 ± 1.06) and within the natural variability between terrestrial samples (7.2−10.9).

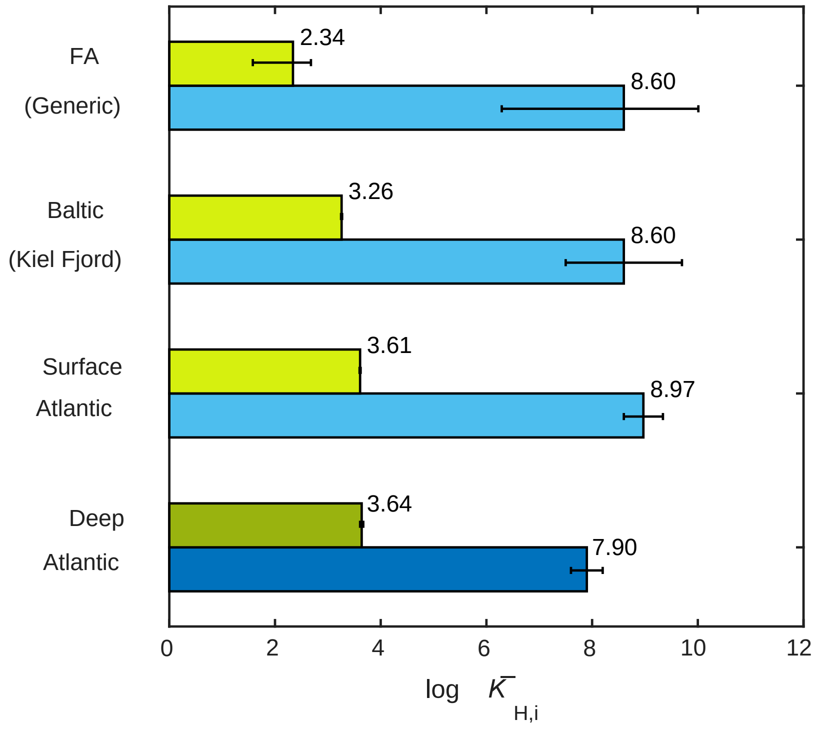

Figure S4. Median values of the intrinsic proton binding affinity ($\log \overline{K}_{H,i}$) parameters obtained from the fits of the standard V_D Donnan model to proton binding data shown in Figure S2 for the DOM_1 (green bars) and DOM_2 (blue bars) distributions. Bar heights indicate the mean, and error bars indicate the standard deviation. The values Baltic Kiel Fjord are from Lodeiro *et al*.[5] Bar heights indicate the mean, and error bars indicate the standard deviation. The values FA (Generic) indicate the generic fulvic acid parameters from Milne *et al*.[7]; the error bar indicates the range of values for the datasets analyzed.

We also calculated the intrinsic ion binding heterogeneity for North Atlantic solid-phase extracted dissolved organic matter (SPE−DOM) based on the standard V_D Donnan model and found values for the low proton affinity groups that are ca. 10% more homogeneous than a coastal DOM, and 35 % more homogeneous than a generic fulvic acid of terrestrial origin (Figure S5). The DOM_1 distribution of the deep open ocean SPE−DOM presents an intrinsic binding heterogeneity of 0.81 ± 0.01, which is close to the maximum value of 1 expected for a perfect homogeneous ligand.

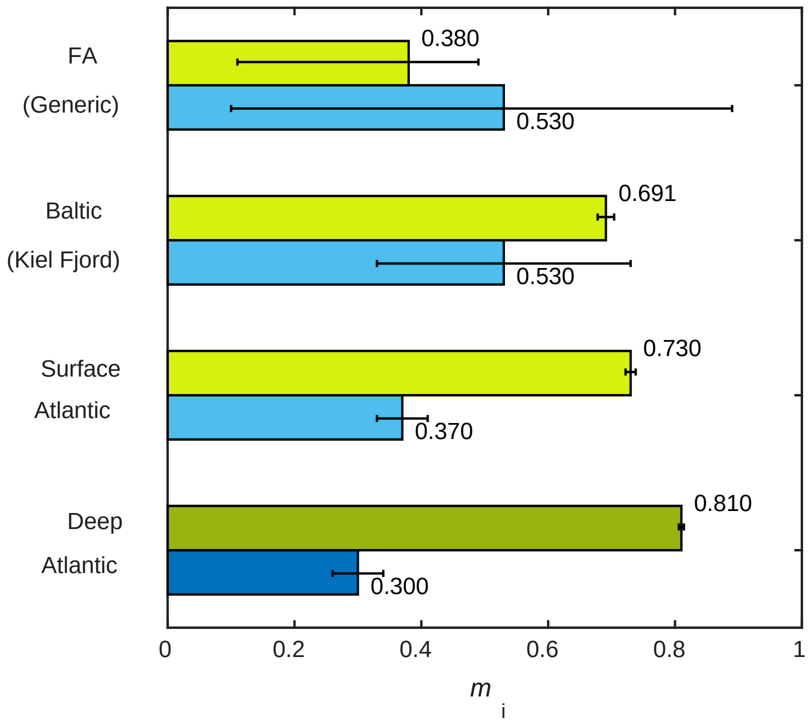

Figure S5. Width of the affinity distribution function (chemical binding heterogeneity, m_i) parameters obtained from the fits of the standard V_D Donnan model to proton binding data shown in Figure S2 for the DOM_1 (green bars) and DOM_2 (blue bars) distributions. The values for the Baltic (Kiel Fjord) are from Lodeiro *et al.*[5] Bar heights indicate the mean, and error bars indicate the standard deviation. The values FA (Generic) indicate the generic fulvic acid parameters from Milne *et al.*[7]; the error bar indicates the range of values for the datasets analyzed.

The intrinsic binding heterogeneity for these low affinity groups in the surface sample is slightly lower (0.73 ± 0.01), and closer to the previously obtained value of 0.69 for a coastal DOM sample. In contrast, the intrinsic *m* value of the high affinity DOM_2 groups in the deep sample (0.30 ± 0.04) was lower, so more heterogeneous, than that observed in the surface sample (0.37 ± 0.04), and both deep and surface samples present a low affinity distribution that is more heterogeneous than the obtained for a generic fulvic acid and a coastal SPE−DOM (0.53).

6. Glossary of terms

Acid-base properties: Proton binding properties.

Charge curve: Representation of the electrostatic charge of DOM ($q_{\mathrm{DOM}} = Q_{\mathrm{maxH,tot}} - Q_{\mathrm{H}}$), obtained from potentiometric titration data using mass and charge balance relationships, *vs* pH.

DOM: Dissolved organic matter.

$\mathbf{DOM_1}$: First main group in the affinity distribution of binding sites, which corresponds to the most acidic binding sites. Usually associated with carboxylic-like functional groups.

$\mathbf{DOM_2}$: Second main group in the affinity distribution of binding sites, which corresponds to the less acidic binding sites. Usually associated with phenolic-like functional groups.

Donnan model: electrostatic model for the description of polyelectrolytic effects, i.e., non-specific binding.

I: Ionic strength.

Intrinsic binding parameters: Binding parameters that are thermodynamically consistent and independent from the specific conditions of the sample.

$\bar{K}_{\mathrm{H},j}$: The median value of the jth affinity distribution for protons, also known as the proton binding affinity.

Master curve: Representation of the electrostatic charge of DOM ($Q_{\mathrm{maxH,tot}} - Q_{\mathrm{H}}$), obtained from potentiometric titration data using mass and charge balance relationships, *vs* $\mathrm{pH_D}$, the local concentration of protons in the Donnan volume (gel phase).

m_j ($0 < m_j \leq 1$): Represents the ion (chemical) binding heterogeneity. It is related to the width of the affinity distribution function. The limiting value of $m_j = 1$ corresponds to a perfectly homogeneous set of sites.

NICA model: Non-Ideal Competitive Adsorption model that describes the chemical binding to heterogeneous ligands

PB-V_D: Expression for V_D consisted with the non-linear Poissson-Boltzman (NLPB) equation.

pH (-log c_H): The local concentration of protons in the bulk solution in the free proton concentration scale.

pH_D (-log $c_{H,D}$): The local concentration of protons in the Donnan volume (gel phase). It is related to its concentration in bulk solution, c_H, by the Donnan factor.

Proton binding affinity spectra: The fraction of binding sites with a specific value of log $\overline{K}_H$. It can be also defined as the density of probability of proton binding affinity.

Q_H: Amount of bound protons per mol of DOC (mmol·mol C^{-1}).

$Q_{maxH,j}$: Total amount of titratable proton binding sites within each *j* distribution (DOM_1 or DOM_2).

V_D: Volume of the permeable gel phase, also known as Donnan volume. Represents the amount of water associated with the polymer (DOM) phase.

References

(1) Lauvset, S. K.; Lange, N.; Tanhua, T.; Bittig, H. C.; Olsen, A.; Kozyr, A.; Álvarez, M.; Becker, S.; Brown, P. J.; Carter, B. R.; Cotrim da Cunha, L.; Feely, R. A.; van Heuven, S.; Hoppema, M.; Ishii, M.; Jeansson, E.; Jutterström, S.; Jones, S. D.; Karlsen, M. K.; Lo Monaco, C.; Michaelis, P.; Murata, A.; Pérez, F. F.; Pfeil, B.; Schirnick, C.; Steinfeldt, R.; Suzuki, T.; Tilbrook, B.; Velo, A.; Wanninkhof, R.; Woosley, R. J.; Key, R. M. *An Updated Version of the Global Interior Ocean Biogeochemical Data Product, GLODAPv2.2021*; preprint; Oceanography – Chemical, 2021. https://doi.org/10.5194/essd-2021-234.

(2) Measures, C.; Hatta, M.; Fitzsimmons, J.; Morton, P. Dissolved Al in the Zonal N Atlantic Section of the US GEOTRACES 2010/2011 Cruises and the Importance of Hydrothermal Inputs. *Deep Sea Res. Part II Top. Stud. Oceanogr.* **2015**, *116*, 176–186. https://doi.org/10.1016/j.dsr2.2014.07.006.

(3) Schlosser, C.; Klar, J. K.; Wake, B. D.; Snow, J. T.; Honey, D. J.; Woodward, E. M. S.; Lohan, M. C.; Achterberg, E. P.; Moore, C. M. Seasonal ITCZ Migration Dynamically Controls the Location of the (Sub)Tropical Atlantic Biogeochemical Divide. *Proc. Natl. Acad. Sci.* **2014**, *111* (4), 1438–1442. https://doi.org/10.1073/pnas.1318670111.

(4) Rijkenberg, M. J. A.; Middag, R.; Laan, P.; Gerringa, L. J. A.; van Aken, H. M.; Schoemann, V.; de Jong, J. T. M.; de Baar, H. J. W. The Distribution of Dissolved Iron in the West Atlantic Ocean. *PLoS ONE* **2014**, *9* (6), e101323. https://doi.org/10.1371/journal.pone.0101323.

(5) Lodeiro, P.; Rey-Castro, C.; David, C.; Achterberg, E. P.; Puy, J.; Gledhill, M. Acid-Base Properties of Dissolved Organic Matter Extracted from the Marine Environment. *Sci. Total Environ.* **2020**, *729*, 138437. https://doi.org/10.1016/j.scitotenv.2020.138437.

(6) Companys, E.; Garces, J. L.; Salvador, J.; Galceran, J.; Puy, J.; Mas, F. Electrostatic and Specific Binding to Macromolecular Ligands - A General Analytical Expression for the Donnan Volume. *Colloids Surf. -Physicochem. Eng. Asp.* **2007**, *306* (1–3), 2–13. https://doi.org/10.1016/j.colsurfa.2007.01.016.

(7) Milne, C. J.; Kinniburgh, D. G.; Tipping, E. Generic NICA-Donnan Model Parameters for Proton Binding by Humic Substances. *Environ. Sci. Technol.* **2001**, *35* (10), 2049–2059. https://doi.org/10.1021/es000123j.
